# Wildfire immediately reduces nest and adult survival of greater sage-grouse

**DOI:** 10.1038/s41598-023-32937-2

**Published:** 2023-07-06

**Authors:** Emmy A. Tyrrell, Peter S. Coates, Brian G. Prochazka, Brianne E. Brussee, Shawn P. Espinosa, Joshua M. Hull

**Affiliations:** 1 Western Ecological Research Center, U.S. Geological Survey, Dixon Field Station, 800 Business Park Drive, Suite D, Dixon, CA 95620 USA; 2 Department of Animal Sciences, University of California Davis, 2251 Meyer Hall, One Shields Avenue, Davis, CA 95616 USA; 3 Nevada Department of Wildlife, 6980 Sierra Center Parkway, Reno, NV 89511 USA

**Keywords:** Conservation biology, Fire ecology, Population dynamics

## Abstract

Wildfire events are becoming more frequent and severe on a global scale. Rising temperatures, prolonged drought, and the presence of pyrophytic invasive grasses are contributing to the degradation of native vegetation communities. Within the Great Basin region of the western U.S., increasing wildfire frequency is transforming the ecosystem toward a higher degree of homogeneity, one dominated by invasive annual grasses and declining landscape productivity. Greater sage-grouse (*Centrocercus urophasianus*; hereafter sage-grouse) are a species of conservation concern that rely on large tracts of structurally and functionally diverse sagebrush (*Artemisia* spp.) communities. Using a 12-year (2008–2019) telemetry dataset, we documented immediate impacts of wildfire on demographic rates of a population of sage-grouse that were exposed to two large wildfire events (Virginia Mountains Fire Complex—2016; Long Valley Fire—2017) near the border of California and Nevada. Spatiotemporal heterogeneity in demographic rates were accounted for using a Before-After Control-Impact Paired Series (BACIPS) study design. Results revealed a 40% reduction in adult survival and a 79% reduction in nest survival within areas impacted by wildfires. Our results indicate that wildfire has strong and immediate impacts to two key life stages of a sagebrush indicator species and underscores the importance of fire suppression and immediate restoration following wildfire events.

## Introduction

Climatic changes, like rising temperatures and drought, are driving more frequent and severe wildfire events on a global scale^1^. While wildfire is essential in shaping and maintaining many ecosystem processes^2^, increases in the duration, frequency, and size of wildfires^3^ threatens to transform many ecological systems. Faunal specialists are particularly vulnerable to rapid landscape level changes because they often exhibit relatively inflexible sets of behaviors that prevent them from adapting at a pace that matches their altered environment^4^. Populations of greater sage-grouse (*Centrocercus urophasianus*; hereafter sage-grouse) located in the Great Basin of the western U.S. are consistent with this phenomenon, and are expected to continue to decline^5^ under future climate scenarios^6,7^. Moreover, the species’ status as an ecological indicator^8,9^ suggests that declines are of greater significance and a symptom of systemic-scale degradation.

Sage-grouse are an ideal candidate for evaluating wildfire impacts to sagebrush (*Artemisia* spp.) ecosystems because they rely on large, undisturbed, and heterogeneous sagebrush communities to complete a sequence of distinct life stages^10–12^. Reproductive displays often occur on communal breeding grounds (leks) and individuals often return to the same leks over multiple breeding seasons^13–17^. Within a historical context, this behavior likely resulted in higher fitness by increasing an individual’s knowledge of a relatively stable environment^15,18^. However, site fidelity could become a maladaptive behavior following wildfire-induced habitat alterations. For example, the continued use of wildfire-affected landscapes by female sage-grouse for nesting, despite reduced shrub cover and a high abundance of non-native annual grasses, can result in reduced adult^19^ and nest survival^20,21^. When avoidance of fire affected areas does occur, it may be delayed by several years^22^, which could lead to short- and long-term demographic consequences. The life history requirements of sage-grouse combined with the rigidity of their philopatric behavior (tendency to remain or return to the same areas) offers an opportunity to study contemporary ecological impacts of wildfire on sagebrush-dependent wildlife within the Great Basin.

Historically, wildfire activity has helped shape and maintain the vegetation component of the Great Basin, producing heterogeneous landscapes through disturbance and ecological succession^23–25^. However, many areas of the Great Basin have recently experienced substantial declines in the duration between fires^24,26–29^, largely a result of the arrival of pyrophytic invasive annual grasses^30^ such as cheatgrass (*Bromus tectorum*), red brome (*B. rubens*), medusahead wildrye (*Taeniatherum caput-medusae*), and ventenata (*Ventenata dubia*). These invasive grasses are reshaping the fire regime through a positive feedback loop^31,32^ that increases fine fuel continuity, reduces spatial complexity, and increases fuel load^5,26,33–35^. Furthermore, the ability of invasive grasses to rapidly reestablish in a post-fire landscape, means that they often outcompete native plant species that have evolved under more moderate fire regimes^36,37^. As a result, the system is advancing toward a higher degree of homogeneity, and landscape productivity is in a state of decline^38^. Managers responsible for administering conservation measures like fire suppression, prevention, and restoration could help reverse trends, and may find success in directing finite resources to areas or populations that express the greatest need. Specific to sage-grouse, it is critical to understand which vital rates are most adversely affected by wildfire. Unfortunately, the literature on sage-grouse response to wildfire is relatively sparse to date and the studies that do exist often lack a spatial or temporal control^19,39,40^. Thus, information on the existence and quality of pre-fire systemic heterogeneity is missing and the absolute effect of wildfire on sage-grouse populations is unknown. To help fill that information gap, we utilized a long-term monitoring dataset^41^ that captured the response of sage-grouse following two wildfire events (the 2016 Virginia Mountains Fire Complex and the 2017 Long Valley Fire; hereafter VMFC and LVF, respectively) that occurred within the Virginia Mountains study area between 2008 and 2019^42^. We chose to evaluate changes in nest and adult survival using a robust Before-After Control-Impact Paired Series (BACIPS) design^43^ because those vital rates are crucial to population maintenance and growth^44–46^.

## Methods

### Study area

**Figure 1:**
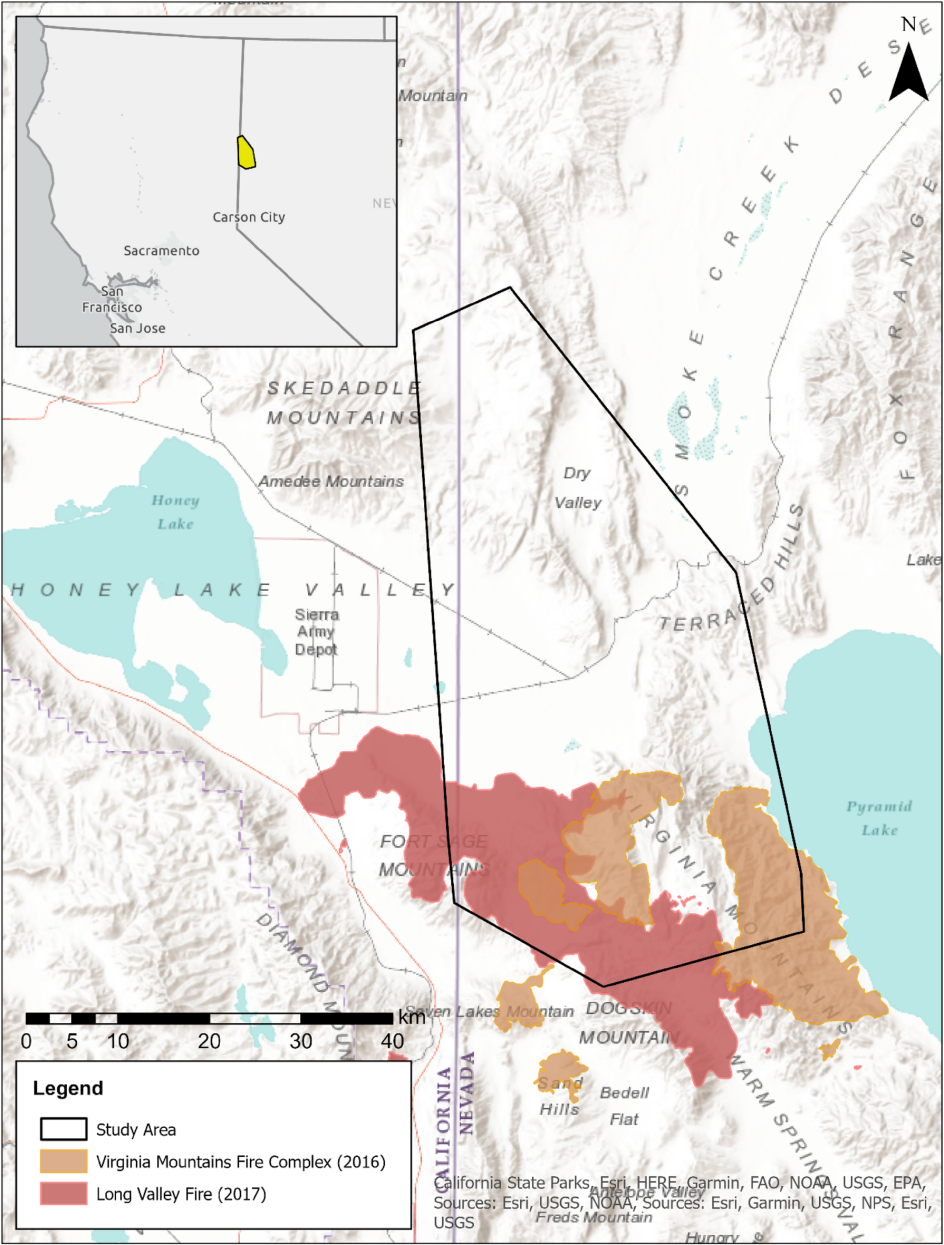
Map of greater sage-grouse (*Centrocercus urophasianus*) study site outlined in black in northwestern Washoe County, Nevada, USA and southeastern Lassen County, California, USA, during 2008–2019. The orange polygon indicates the extent of the 2016 Virginia Mountains Fire Complex. The red polygon indicates the extent of the 2017 Long Valley Fire. Fire perimeters were downloaded from the Monitoring Trends in Burn Severity (MTBS) website^42^.

The Virginia Mountains study area encompasses 123,607 ha bordering northwestern Nevada (Washoe County), USA and northeastern California (Lassen County), USA and occupies the Basin and Range Province, which is a physiographic region characterized by an alternating pattern of generally north–south oriented valleys and mountain ranges (Fig. 1). The study area falls within the rain shadow of the Sierra Nevada Mountain Range, and experiences semi-arid climatic conditions that are consistent with the rest of the Great Basin; specifically dry, hot summers and cold wet winters. The vegetation communities within the study area can be categorized by three distinct zones which include salt desert shrubland (low elevation), pinyon-juniper (*Pinus monophylla*, *Juniperus osteosperma*) woodland (mid-elevation), and mountain shrub (high-elevation). Between 2008 and 2019 the study area experienced two independent wildfires over two consecutive years (2016–2017)^42^. The VMFC started on 28 July 2016 and consisted of five geographically isolated, but proximal fires, all of which originated from lightning strikes during a single storm system. The total area burned during the VMFC was 27,171 ha with 22,282 ha occurring within the study area boundary (18.0% of total study area). The LVF started on 11 July 2017 and consisted of a single, contiguous burn, which originated along Highway 395 approximately 40 km northwest of Reno, Nevada. The LVF burned a total of 33,886 ha with 32,585 ha occurring within the study area boundary (26.4% of total study area). The total area burned across both wildfire events was 50,412 ha or 40.8% of the study area (Fig. 1).

### Capture and monitoring of sage-grouse

We captured female sage-grouse between 2008–2019 during the spring (March–April) and fall (September–November) seasons at night using a spotlighting technique^47,48^. Following capture, each bird was fitted with a uniquely numbered aluminum leg-band and necklace-style very high frequency (VHF; Advanced Telemetry Systems, Isanti, MN, USA) radio-transmitter equipped with mortality sensor. VHF collars did not exceed 3.5% of bird body mass (mean = 2.01%, SD = 0.37%). Capture, handling, and marking procedures were approved by U.S. Geological Survey Western Ecological Research Center's Animal Care and Use Committees (WERC-2015-02). Reporting of animals used in analyses follows recommendations from the ARRIVE guidelines^49^.

Radio-marked sage-grouse were monitored according to season (i.e., nesting, brood-rearing, non-breeding) and individual reproductive status (e.g., nesting vs non-nesting). We located individuals once every three days during the nesting season (March–June) to capture nest initiation. Females located within the same area on two consecutive visits were visually inspected to confirm nesting activity. Once confirmed, we continued monitoring nests every three days until fate was determined (i.e. successful, abandoned, depredated). Nests were considered successful if ≥ 1 egg hatched, which was determined by examination of eggshells or the presence of chicks within or near the nest bowl. We categorized nests as unsuccessful if no eggs were present, all eggs showed signs of depredation (e.g. holes in the side of eggshells, membrane still attached to eggshell), or intact eggs failed to hatch and the female did not return (i.e. abandoned). We continued to monitor female sage-grouse once a week following nest failure to confirm possible re-nesting attempts. Individuals that successfully hatched ≥ 1 egg were monitored up to 50-d post-hatch at 10-d intervals. Monitoring of females with broods ceased once fate was determined (i.e., failed or fledged). Individuals that were no longer nesting or rearing broods were monitored once per week until the fall season (early September) at which point locations were acquired at 1–2-mo intervals using fixed-wing aircraft (i.e. aerial telemetry). Aerial telemetry was conducted during spring and summer months (March–August) to aid ground crews when workload or access curtailed timely relocation of radio-marked birds. Accuracy of aerial telemetry was assessed using stationary collars of known location, and never exceeded 200 m. Telemetry effort was normally distributed across the year with the bulk of locations occurring between April–July. This period is associated with a higher degree of individual-based decision making and a more diffuse population-level space use behavior^50–52^, which necessitates location data of higher temporal resolution where the objective is assessing variation in survival as a function of spatial covariates. Conversely, late fall to early spring is marked by a higher degree of sociality^53–55^, which allows a relaxation of relocation rates and a borrowing of information across individuals using time-varying, population-level parameters to impute missing values (see *Data Analysis: Adult Survival*).

### Study design

For both nest and adult survival we employed a BACIPS design^43^ within a Bayesian framework using nest monitoring and telemetry data collected before and after wildfire events within impact and control areas respectively (see *Analysis 1 and Analysis 2* below)^41^. The purpose of the BACIPS analysis was to quantify differences in nest and adult survival across the four BACIPS groups to yield a relative effect of wildfire on nest and adult survival. We used wildfire data downloaded from the Monitoring Trends in Burn Severity (MTBS) website^42^ to generate the four BACIPS groups which served as a categorical covariate depicting the spatiotemporal relationship of each bird relative to the VMFC and LVF. Values were restricted to the range of positive integers 1–4 and represented, in order, the four BACIPS groups associated with wildfire (before-control = BC, before-impact = BI, after-control = AC, after-impact = AI). Categorical periods (before vs. after) represent the position of a location in time relative to a wildfire event, whereas categorical treatments (impact [inside] vs. control [outside]) represent the position of a location in space relative to a wildfire perimeter.

### Data analysis

#### Nest survival

We conducted a BACIPS study design within a Bayesian framework to estimate the effects of the VMFC and LVF using nest monitoring data collected at the Virginia Mountains study area from 2008 to 2019. We generated encounter histories for each sage-grouse nest (n = 75 pre-wildfire; n = 34 post-wildfire) using the date the nest was found, last date observed active, and date that nest fate (i.e. successful or unsuccessful) was determined. Nests that failed between the initial nest location (i.e. first found) and the second nest check were assumed to survive one day, which allowed us to retain these instances and avoid inflating survival estimates. For nests, the BACIPS consisted of concurrently monitoring nests before and after the wildfire event within (impact) and outside (control) of the wildfire perimeters. Nests were assigned to each respective treatment group (control or impact) by overlaying nest locations with the VMFC and LVF perimeters^42^.

We used Bayesian shared frailty models^56,57^ to estimate a daily unit hazard (*UH*) for each nest. We specified separate baseline log-hazards (${\alpha }_{g}$) for each BACIPS group (g). Additional parameters included log-hazard ratios for age class (${\beta }_{c}$) and bird (${\gamma }_{b}$), which were related to the daily unit hazard (*UH*) through the expression:

$${UH}_{id}=\mathit{exp}\left({\alpha }_{g}+{\beta }_{c}+ {\gamma }_{b}\right),$$

$${\alpha }_{g} \sim Uniform\left(-20, 0\right),$$

$${\beta }_{c} \sim Normal\left(0, 30\right),$$

$${\gamma }_{b} \sim Normal \left(0, {\sigma }_{\gamma }\right),$$

1 $${\sigma }_{\gamma } \sim Uniform\left(0, 20\right).$$

Subscripts *i* and *d*, reference nest and day, respectively.

The cumulative probability of nest survival (${S}_{i}$) for each nest (*i*) took the form:

2 $${S}_{i}={e}^{{-CH}_{i}},$$

where:

3 $${CH}_{i}= \sum_{1}^{T}{UH}_{1:d,i},$$

represents the cumulative hazard (*CH*) function. The discrete outcome (${y}_{i}$) for each nest, coded 1 (successful) or 0 (failed), served as the response variable, and ${S}_{i}$ the probability of survival:

4 $${y}_{i}\sim Bernoulli\left({S}_{i}\right).$$

Posterior distributions of nest survival (${S}_{i}$) were derived for each BACIPS group using parameters ${\alpha }_{g}$ and ${\beta }_{c}$. We based inference of nest survival on a 37-d ($T$) egg-laying and incubation period^39^.

Posterior distributions of parameters were estimated using Markov chain Monte Carlo (hereafter MCMC) simulations from three chains of 10,000 iterations thinned by a factor of 10 following a burn-in of 50,000 iterations. Models were run in program JAGS version 4.3^58^ through R version 3.4.0^59^ using RStudio version 1.2.5042^60^. We did not find a lack of convergence among any of the parameters monitored (R-hat < 1.1). We present median values of estimated parameters and 95% credible intervals unless otherwise specified.

We evaluated evidence of effects of wildfire using published BACIPS ratio methods^43^ and CI-divergence measures^61^ applied to the posterior distributions generated from our frailty model. Using a Bayesian approach to calculate BACIPS ratios provides test statistics that compute positive or negative effects^43^. To evaluate wildfire treatment (i.e. impact or control) effects for this BACIPS study, we used the posterior distributions of nest survival probabilities ($${S}_{i})$$ to derive ratios ($${{R}_{S}}_{p,i|c})$$ of impact to control (*i|c*) for each period (*p*), where *p* references periods before (*b*) and after (*a*) the wildfire:

5 $${{R}_{S}}_{p,i|c}= \frac{{S}_{p,i}}{{S}_{p,c}}.$$

We then estimated the posterior distribution of the mean treatment effect for nest survival:

6 $${R}_{{S}_{BACIPS}}=\frac{{\overline{R} }_{{S}_{i|c\, after}}}{{\overline{R} }_{{S}_{ i|c\, before}}}.$$

We also calculated two control-impact (CI) measures. CI-contribution quantifies by what extent the change between periods is stronger (i.e. greater change within impacted sites or control sites). CI-divergence quantifies the degree to which control and impact sites have diverged between the after and before period^61^. CI-contribution and CI-divergence were calculated as follows:

$$\mathrm{CI}-\mathrm{contribution }= \left|{S}_{i \,after}-{S}_{i \,before}\right|-\left|{S}_{c \,after}-{S}_{c \,before}\right|,$$

7 $$\mathrm{CI}-\mathrm{divergence }= \left|{S}_{i \,after}-{S}_{c \,after}\right|-\left|{S}_{i \,before}-{S}_{c \,before}\right|.$$

For CI-contribution, positive values demonstrate a greater change in impacted areas relative to control areas, whereas negative values reflect greater changes in control areas. For CI-divergence, positive values reflect greater variability between impact and control areas following wildfire, while negative values reflect decreased variability following wildfire. By using these CI measures combined with BACIPS ratios we were able to conduct a comprehensive investigation of wildfire impacts on nest survival.

#### Adult survival

**Figure 2:**
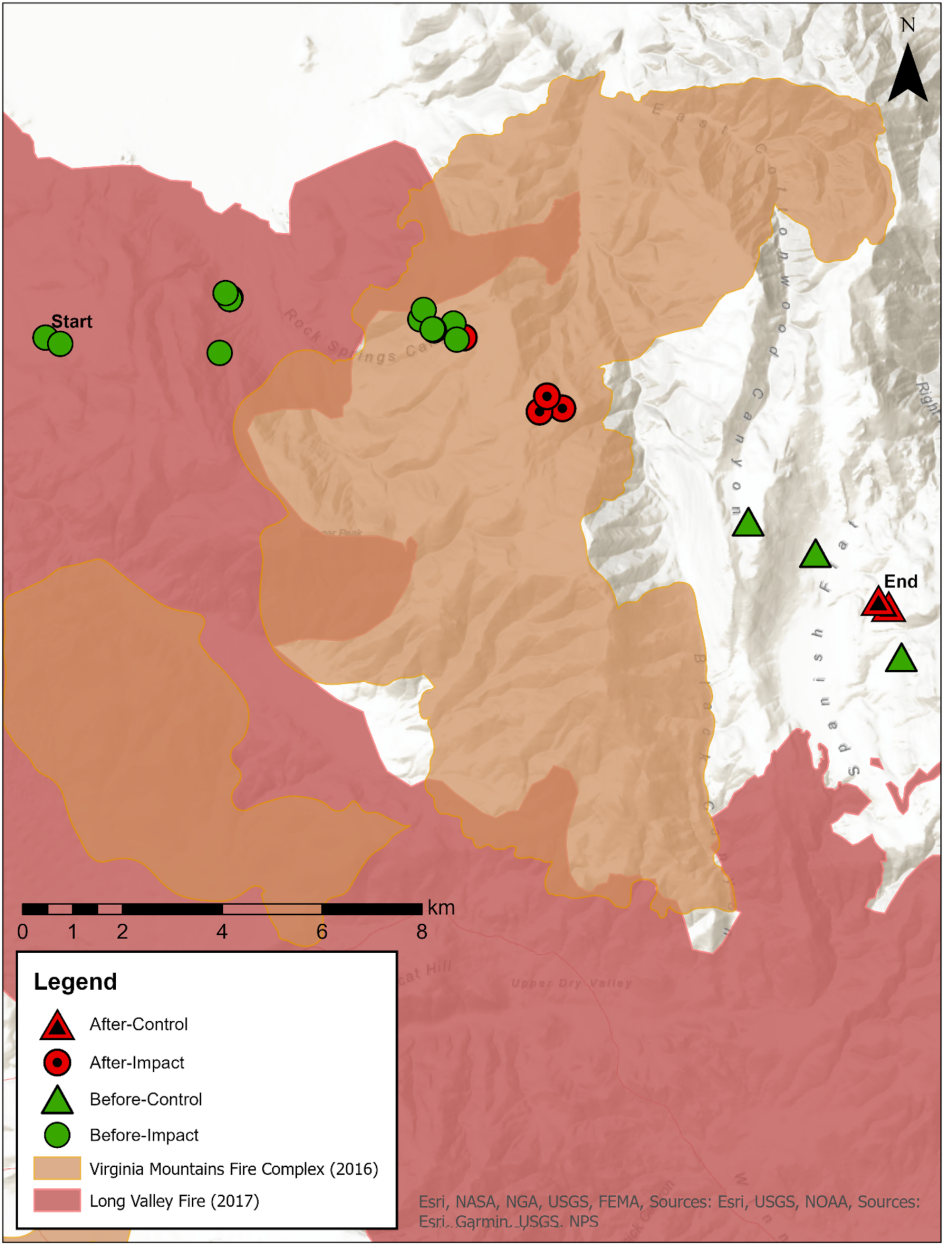
Map of individual female sage-grouse (*Centrocercus urophasianus*) traveling through each of the four before-after-control-impact (BACIPS) groups within the Virginia Mountains before and after the 2016 Virginia Mountains Fire Complex and 2017 Long Valley Fire.

Unlike nests, adults can change BACIPS group assignment based on their location in space and time (Fig. 2). This quality necessitated the use of a spatiotemporally varying BACIPS covariate. Thus, we generated monthly encounter histories for adult sage-grouse which included information about the month the individual was first located, the last month it was observed alive, and the month it left the study (i.e. mortality or lost). For each month of the encounter history, individuals were assigned a BACIPS group by overlaying their location data with the VMFC and LVF perimeters^42^. Individuals that were relocated ≥ 2 times within the same month had the opportunity to occupy more than one BACIPS group. A simple decision tree was constructed to handle these events and was predicated upon a hierarchy of assumed impact to survival wherein the after group took precedence over the before group, the impact group took precedence over the control group, and the treatment group took precedence over the period group. Because a relatively small proportion of the dataset required the application of the decision tree (~ 9.3%), an alteration in the hierarchy is suspected to have had minimal impact to results.

Within the adult survival analysis, additional time-varying covariates were specified for age class and month based on a priori knowledge of potential confounding effects that could arise from variation in survival associated with sage-grouse life histories^44,62^. The time-varying covariate for age class allowed yearling sage-grouse to graduate into the adult age class on 1-March of each year and remain there for the duration of the study. Sage-grouse captured as adults remained in the adult age class for the duration of the study. The use of a time-varying covariate for month allowed us to account for temporal variability in survival across a year^62^. This technique was necessary to account for potential sources of bias because of uneven sampling (i.e. captures) among spring and fall seasons across years, and the variability in survival across seasons.

We used a Bayesian shared frailty model^56,57^, similar to that shown in Eqs. (1, 2, 3 and 4), to estimate cumulative annual adult survival as a continuous process observed at discrete, monthly intervals. The Bayesian and shared frailty frameworks were particularly advantageous for this analysis given the study design and treatment of missing values. Working within a Bayesian framework allowed us to treat missing values as parameters in our model and assign a prior distribution to each parameter. In the case of the BACIPS time-varying covariate, we assigned a categorical distribution with the hyperparameter π, which represented an M x N matrix of non-negative probability weights. Here, the rows of our probability weight matrix represented study intervals and columns the proportion of individuals observed occupying each BACIPS category. This prior specification was chosen based on the seasons which experienced the greatest degree of data sparsity (i.e. winter) and the social nature of sage-grouse during that period^53,55^. We also included a treatment-period interaction term similar in nature to previous BACIPS studies^63,64^. Aside from the adoption of time-varying covariates and a monthly log-hazard ratio, our adult and nest survival models were identical in structure.

We sampled 100,000 iterations from our adult survival model using three independent chains following a burn-in of 50,000 iterations per chain. Posterior samples saved for inference were thinned by a factor of five. Convergence diagnostics and BACIPS ratio calculations followed methods described for nest survival output.

## Results

### Nest survival

**Figure 3:**
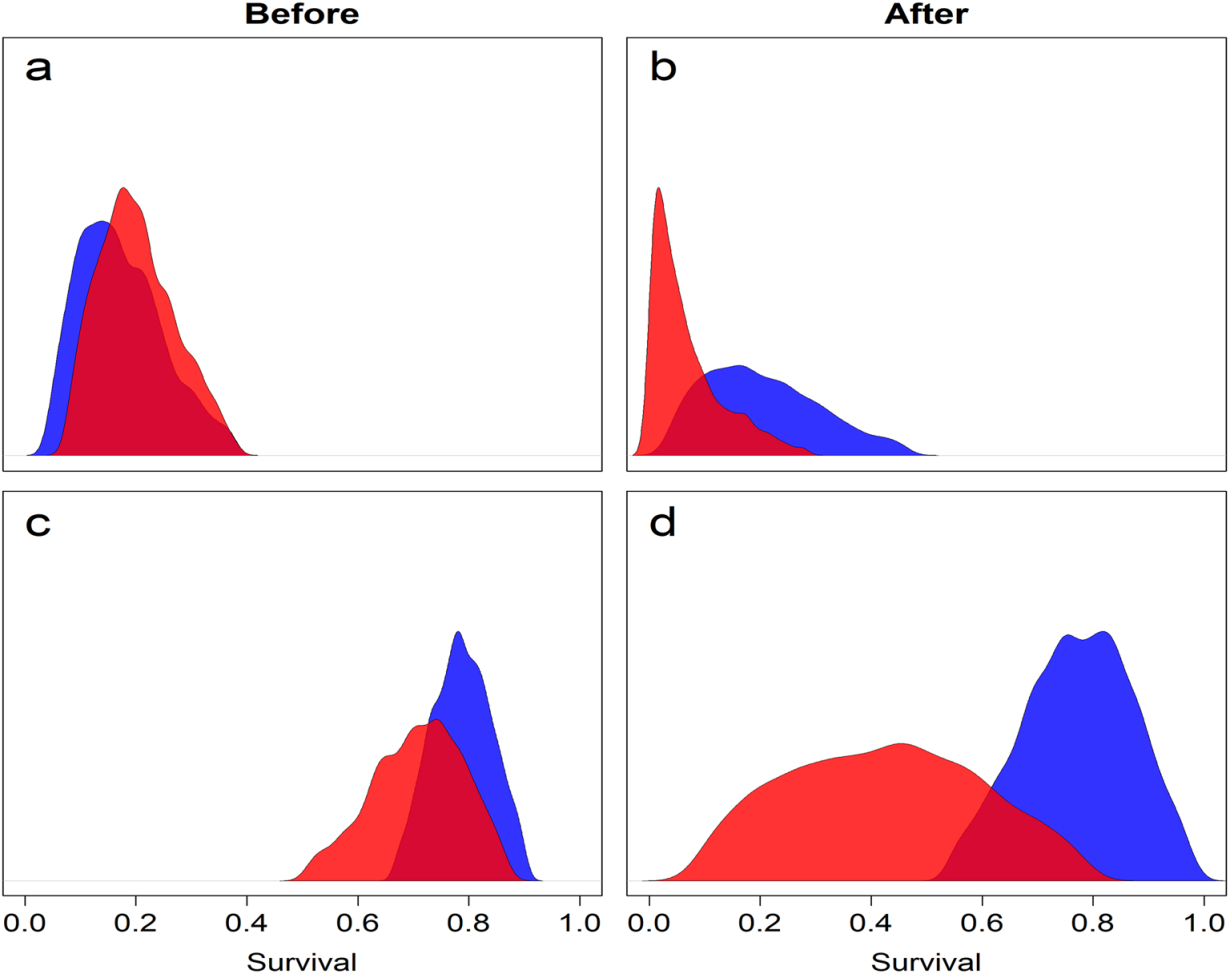
Estimated cumulative nest (panels (**a**) and (**b**)) and adult (panels (**c**) and (**d**)) survival probabilities for greater sage-grouse (*Centrocercus urophasianus*) in Washoe County, NV, and Lassen County, CA, USA, before (2008–2016) and after (2017–2019) the 2016 Virginia Mountains Fire Complex and 2017 Long Valley Fire. Blue distributions represent average cumulative survival probabilities for control (unburned-before and unburned-after) areas. Red distributions represent average cumulative survival probabilities for impacted (burned-before and burned-after) areas.

**Figure 4:**
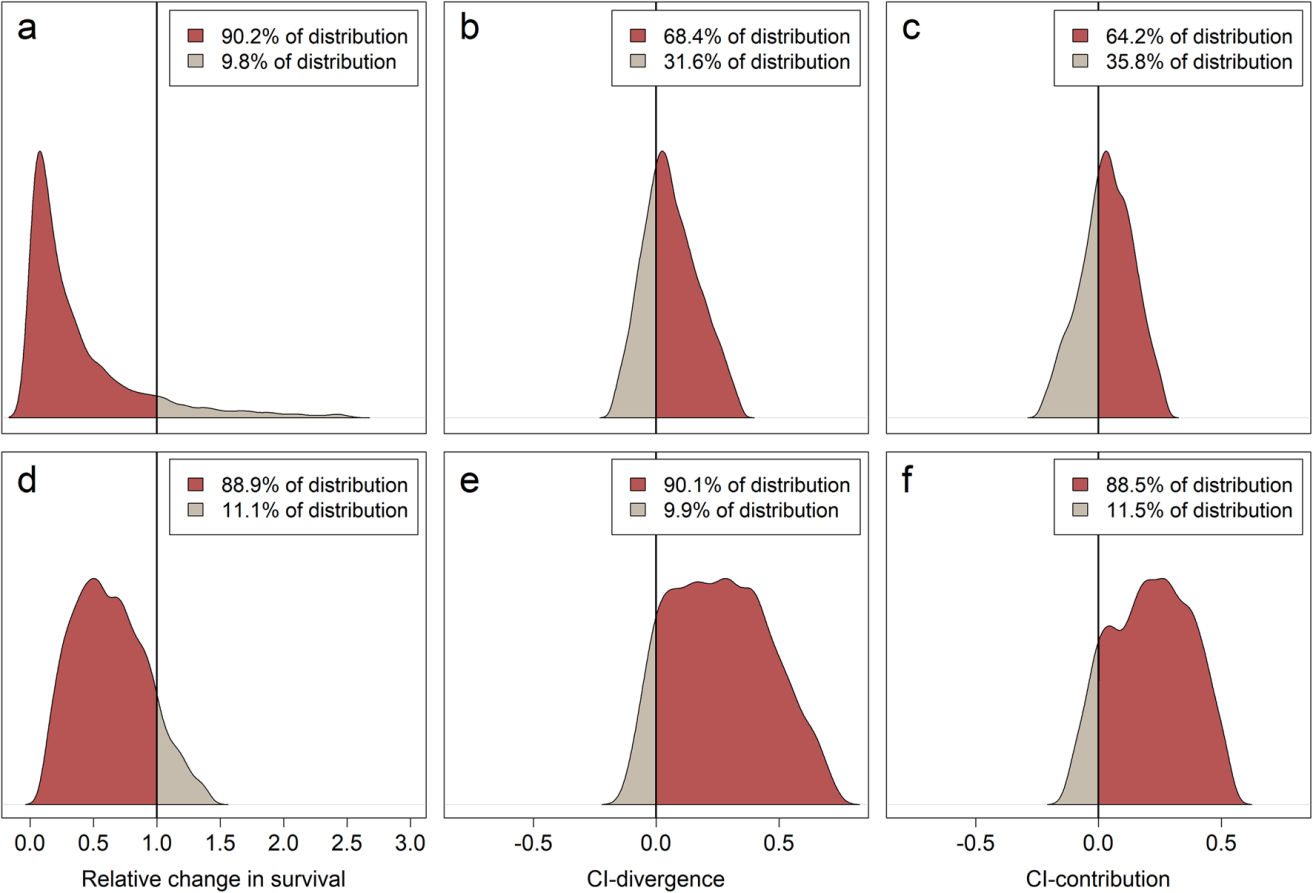
Distribution of BACIPS (before-after control-impact paired-series) ratios (panels (**a**) and (**d**)) calculated from cumulative survival probabilities of greater sage-grouse (*Centrocercus urophasianus*) nests (panels (**a–c**)) and adults (panels (**d–f**)) in Washoe County, NV, and Lassen County, CA, USA based on impacts from the 2016 Virginia Mountains Fire Complex and 2017 Long Valley Fire. Samples from the posterior distribution that are below a value of one (vertical line) represent evidence for a decrease in cumulative survival probability in impact areas relative to control areas after wildfire. Samples from the posterior distribution of CI-divergence (panels (**b**) and (**e**)) that are above zero represent evidence for greater divergence in survival between impact and control areas following wildfire relative to the same areas before wildfire. Samples from the posterior distribution of CI-contribution (panels (**c**) and (**f**)) that are above zero represent evidence for a greater degree of change in survival within impact areas (after relative to before wildfire) compared to changes in survival within control areas over the same time.

Using a BACIPS framework, we investigated the differences in survival probabilities among 75 pre-wildfire nests (31 before-control, 44 before-impact) and 34 post-wildfire nests (18 after-control, 16 after-impact). Nest survival probabilities within control sites were 0.16 (95% CRI = 0.04–0.38) before wildfire and 0.19 (95% CRI = 0.03–0.46) after wildfire (Table 1; Fig. 3a). Nest survival probabilities within impacted areas were 0.19 (95% credible interval [CRI] = 0.08–0.38) before wildfire and 0.05 (95% CRI = 0.00–0.28) after wildfire (Table 1; Fig. 3b). During the same period that fire-affected sites experienced a decrease in survival, fire-unaffected areas experienced an increase. A comparison of survival ratios (impact:control) revealed that survival probabilities within impacted areas were 1.20 times (95% CRI = 0.36–4.60) their control counterparts during the before period and 0.26 (95% CRI = 0.01–2.58) times controls post fire. These ratios produced a BACIPS ratio of 0.21 (95% CRI = 0.01–2.50, Table 2), meaning that nest survival within burned areas decreased by 79% relative to unburned areas, post fire. Although the upper CRI of the BACIPS parameter incorporated values greater than 1, suggesting a positive fire effect, this was largely due to the right skew of the posterior distribution. A summary of posterior samples revealed that 90.2% of the probability distribution fell below a value of 1 (Fig. 4a). Simply put, models indicated > 90% chance that nest survival decreased following wildfire within the impact area, and < 10% chance that it increased, relative to controls. Median estimates of CI-divergence (0.05; 95% CRI = -0.17–0.34, Table 2) were slightly above a value of 0, indicating that control and impact sites were more dissimilar after wildfire compared to before (Fig. 4b). Similarly, median estimates of CI-contribution values were slightly above 0 (0.04; 95% CRI = − 0.23–0.27, Table 2), suggesting that the change in survival across periods was greater among impact groups (i.e., before-impact, after-impact) than control groups (i.e. before-control, after-control) in the wake of wildfire (Fig. 4c).

**Table 1:** Estimated cumulative survival probabilities (median and 95% credible interval; CRI) of greater sage-grouse (Centrocercus urophasianus) nests and adults in Washoe County, NV, and Lassen County, CA, USA before (2008–2016) and after (2017–2019) the Virginia Mountains Fire Complex and Long Valley Fire, outside (control) and inside (impact) the fire perimeter.

Vital rate	Temporal spatial group	n	Survival probability	95% CRI
Nest survival	Before-control	31	0.16	(0.04–0.38)
	Before-impact	44	0.19	(0.08–0.38)
	After-control	18	0.19	(0.03–0.46)
	After-impact	16	0.05	(0.00–0.28)
Adult survival	Before-control	453	0.79	(0.67–0.90)
	Before-impact	487	0.71	(0.50–0.87)
	After-control	325	0.77	(0.54–0.99)
	After-impact	139	0.42	(0.08–0.79)

Sample sizes (n) reflect the number of nests (Nest survival) or telemetry relocations (Adult survival).

**Table 2:** Estimated median and 95% credible interval (CRI) of relative change (BACI), CI-contribution and CI-divergence measures of relative change of cumulative nest and adult survival probabilities of greater sage-grouse (Centrocercus urophasianus) contrasting impact and control areas in Washoe County, NV, and Lassen County, CA, USA, from 2008–2019.

Vital rate	Parameter	Median	95% CRI
Nest survival	BACIPS-relative change	0.21	(0.01–2.50)
	CI-contribution	0.04	(− 0.23–0.27)
	CI-divergence	0.05	(− 0.17–0.34)
Adult survival	BACIPS-relative change	0.60	(0.12–1.40)
	CI-contribution	0.22	(− 0.12–0.54)
	CI-divergence	0.26	(− 0.11–0.72)

### Adult survival

We estimated yearling and adult survival probabilities in relation to wildfire affected areas using 178 radio-marked, female sage-grouse. Due to the relatively long-lived and mobile nature of sage-grouse, it is difficult to summarize the number of individuals belonging to each BACI group. However, the percent of relocations recorded within each of the four BACI groups was 32.2% (before-control), 34.7% (before-impact), 23.1% (after-control), and 9.9% (after-impact). Survival probabilities were higher among individuals occupying control sites during the entire duration of the study. However, greater disparity among control and impact areas was observed, post-fire. During pre-fire periods control sites exhibited survival probabilities around 0.79 (95% CRI = 0.67–0.90; Fig. 3c), while impacted areas were approximately 8% lower on average (0.71; 95% CRI = 0.50–0.87; Table 1). Within the post-fire landscape, control sites remained fairly consistent with a survival probability of 0.78 (95% CRI = 0.54–0.99; Fig. 3c). However, fire-affected areas exhibited a marked drop in survival with median estimates around 0.42 (95% CRI = 0.08–0.79; Table 1, Fig. 3d). Prior to wildfire events, the survival ratio of impact to control ( ${\overline{R} }_{{S}_{i|c\, before}}$) was 0.91 (95% CRI = 0.64–1.14). In the wake of wildfire, the same statistic dropped to 0.55 (95% CRI = 0.09–1.21, Table 1). Using before and after ratios, we calculated a BACIPS ratio which resulted in an overall median wildfire effect of 0.60 (95% CRI = 0.12–1.40; Table 2), which indicates that adult survival within burned areas decreased approximately 40% relative to unburned areas over the same time interval. A summary of the posterior distribution of the adult survival BACIPS ratio revealed strong evidence for a negative effect of wildfire. Specifically, models indicated an 88.9% chance that adult survival probabilities were lower within impacted areas, compared to controls, following wildfire (Fig. 4d). CI-measures for adult survival produced similar, albeit stronger, relationships as our nest survival results. Median estimates of CI-divergence (0.26; 95% CRI = − 0.11–0.72; Table 2, Fig. 4e) and CI-contribution (0.22; 95% CRI = − 0.12–0.54; Table 2, Fig. 4f) indicated that control and impact sites were more dissimilar after wildfire in comparison to before and that the change between before and after wildfire in impacted areas was stronger than control areas, respectively.

## Discussion

Sagebrush ecosystems within the Great Basin are facing increased rates of change driven by invasive grass-wildfire cycles^29,30^. Combined with increasing anthropogenic disturbance and climatic shifts, further fragmentation from habitat loss and/or degradation threatens biodiversity of the region^65,66^. Specialist species, like sage-grouse^67^, may face higher risks of localized extirpation if they are unable to adapt to rapid landscape level changes^68^. Our results provide compelling evidence that wildfire adversely impacts adult and nest survival in sage-grouse; life stages important to population maintenance and growth^44–46^. To our knowledge, this study represents the first attempt to evaluate wildfire effects using time varying BACI categorizations that change with an individual's location. It is also one of the first BACIPS studies to capture acute wildfire effects on sage-grouse nest survival using an uninterrupted time series of monitoring data. These data and modeling features allowed us to describe discrepancies between control and impact groups using pre-wildfire data, and to distinguish natural fluctuations in demographic rates from wildfire effects. Findings not only corroborate broad scale analyses that identified links between wildfire and sage-grouse population growth^5^, but provide a demographic basis for the observed influences of wildfire on patterns in growth.

Annual productivity and population growth for sage-grouse has been found to be influenced by variation in both nest and adult survival, with impacts to population maintenance^44^. While we did not directly measure impacts of wildfire on population growth, we found nest survival decreased by 79% in impacted areas relative to controls, while adult survival decreased by 40%. Disturbances, such as wildfire, that have impacts across multiple life stages likely have compounding influences on population growth, indicating that this population likely experienced substantial declines resulting from wildfire. Population growth rates have also been linked strongly to annual changes in brood survival^44^. Sage-grouse chicks may benefit from successional growth of forbs immediately following wildfire, and in burned regions resistant to annual grass invasion, potentially resulting in positive impacts to brood survival. Increased brood survival following wildfires may initially offset negative effects observed in other life stages, leading to minimal disruption to population growth rates immediately following wildfire. However, because sagebrush ecosystems require long recovery times, especially within drier and warmer low- to mid-elevation sagebrush communities, adverse impacts of wildfire on population growth likely worsen over time as state transition from shrubland to annual grassland results in decreased survival across multiple life stages^20,69^, including broods. Therefore, it is important to understand both the immediate and potential chronic effects that wildfire might have on sage-grouse demographic rates and population growth.

Conversion of shrublands to annual grasslands not only results in more persistent impacts of wildfire on sage-grouse, but also changes to fire regimes, namely more frequent and severe wildfires^26,70^. Exotic grasses outcompete native grasses and forbs^32^ and their establishment increases fuel availability leading to greater likelihood for repeat fires within an area^26,70^. Our results indicated lower adult survival before the VMFC and LVF within areas that burned (i.e., before-impact) compared to those that did not (i.e., before-control), suggesting the areas may have previously experienced habitat degradation. A previous wildfire occurred within this study area during 1999 (i.e. Fish Springs fire), and largely overlapped fire perimeters from the VMFC and LVF. Thus, the low pre-fire estimates of adult survival observed in this study may indicate the existence of chronic impacts of wildfire. Furthermore, our pre-wildfire estimates of nest survival align with post-wildfire estimates observed from another study of sage-grouse nest survival within the Great Basin^71^. Thus, the vital rates observed in this paper likely reflect a combination of both immediate and long-term impacts of wildfire and highlight the amplification of effects that shortened fire cycles can have on sage-grouse populations.

Many studies have addressed the impact of wildfire on sage-grouse habitat finding that wildfire reduces cover needed for nesting and other life stages, increasing the chance of mortality and nest failure^16,39^. Sagebrush, an important forage and cover shrub, is slow to reestablish and is often killed during low intensity fires^37,72^. With over 40% of our study area burned from the VMFC and LVF, important forage and refuge sources were likely impacted. Reduction of vegetation from wildfire may increase detection, leading to not only higher predation rates on adults, but also higher likelihood of nest predation because visual predators like common ravens (*Corvus corax*) can more readily detect female sage-grouse attempting to return to their nest. Recent studies have found sage-grouse that nest within burned areas experience lower nest survival compared to those in unburned areas because of decreased cover^20,73^. Furthermore, a recent study examining post-wildfire effects on adult survival found high mortality for individuals that stayed or returned to the burned area likely because of loss of habitat needed for refuge from predation^40^. Because we did not observe direct fire mortality of adult birds in this study (e.g., burning, asphyxiation from smoke inhalation) it is inferred that the majority of fitness consequences stem from post-fire utilization. During winter months, sagebrush becomes increasingly important to sage-grouse populations as it represents the primary component in their diet^53,55^. This period is also marked by elevated survival and increases in endogenous reserves. The loss of sagebrush from late season fires combined with large snow years could lead to decreased food availability, which can have immediate impacts on over-winter survival^74,75^ and carry-over effects on body condition leading into the breeding season. Despite the fitness consequences associated with the use of burned areas, we found that sage-grouse are apt to reoccupy areas shortly after wildfire, which is consistent with several other studies^40,73^. The high level of philopatric behavior exhibited by sage-grouse^14^ makes them ill-suited to adapt at the same rate that modern-era landscape alterations are occurring, especially as wildfire frequency and severity increase across the American West.

This study was not without limitations. First, while traditional BACIPS assumes the entire population is either impacted or not (control), we lacked independence between individuals for each treatment group. However, it is important to point out that our design accounted for movement of sage-grouse in and out of impact areas thereby allowing a more powerful approach than traditional BACIPS design. Additionally, our study design limited our ability to research the conditions driving the mechanisms behind the BACIPS results such as changes in sage-grouse habitat. While beyond the scope of this study, additional investigation specifically focused on the underlying mechanisms leading to low survival is warranted. Our study suggests that management decisions may be better guided with further understanding of how wildfire affects the indirect factors which influence sage-grouse predation risk, food availability, and habitat cover.

## Summary

Understanding how wildlife populations respond to landscape disturbances is central to management and conservation decisions. Within the Great Basin, those decisions are being framed around wildfire, which is expected to reduce sage-grouse populations by approximately 43% by 2044^5^. Our study has provided clear, compelling evidence that wildfire adversely impacts the survival and reproductive potential of this sagebrush-obligate and indicator species. Furthermore, results have demonstrated that wildfire has the potential to reduce population growth and maintenance via both short- and long-term mechanisms. For populations that exist on the periphery of the sage-grouse range, like the one studied here, wildfire has the potential to increase extirpation probabilities^67^. As wildfire trends continue, ecologically valuable habitats are likely to become increasingly fragmented and/or degraded^26,68,70,72^, further reducing the ability of populations to self-rescue. Management practices such as wildfire suppression may be of greater importance within drier and warmer low- to mid-elevation sagebrush communities as these areas have slow-to-no recovery potential and are at a higher risk of invasion by pyrophytic annual grasses^76^. Restoration activities (i.e. seeding, sagebrush planting) aimed at replenishing immediate resource needs may represent an alternative option where wildfire suppression is logistically more difficult or less effective when it does occur^77^. By identifying sage-grouse life-stages most affected by wildfire, wildlife managers may better identify management practices which best support sage-grouse populations^78^ and curtail the effects of increasing wildfire within the Great Basin.

## Data Availability

Data and associated metadata are archived and publicly available at the U.S. Geological Survey ScienceBase website (10.5066/P9WA2M2Y).
